# Digested sludge-derived three-dimensional hierarchical porous carbon for high-performance supercapacitor electrode

**DOI:** 10.1098/rsos.172456

**Published:** 2018-04-11

**Authors:** Jia-Jia Zhang, Hao-Xiang Fan, Xiao-Hu Dai, Shi-Jie Yuan

**Affiliations:** State Key Laboratory of Pollution Control and Resource Reuse, College of Environmental Science and Engineering, Tongji University, Shanghai 200092, People's Republic of China

**Keywords:** digested sludge, supercapacitor, three-dimensional hierarchical porous carbon, energy storage

## Abstract

Digested sludge, as the main by-product of the sewage sludge anaerobic digestion process, still contains considerable organic compounds. In this protocol, we report a facile method for preparing digested sludge-derived self-doped porous carbon material for high-performance supercapacitor electrodes via a sustainable pyrolysis/activation process. The obtained digested sludge-derived carbon material (HPDSC) exhibits versatile O-, N-doped hierarchical porous framework, high specific surface area (2103.6 m^2^ g^−1^) and partial graphitization phase, which can facilitate ion transport, provide more storage sites for electrolyte ions and enhance the conductivity of active electrode materials. The HPDSC-based supercapacitor electrodes show favourable energy storage performance, with a specific capacitance of 245 F g^−1^ at 1.0 A g^−1^ in 0.5 M Na_2_SO_4_; outstanding cycling stability, with 98.4% capacitance retention after 2000 cycles; and good rate performance (211 F g^−1^ at 11 A g^−1^). This work provides a unique self-doped three-dimensional hierarchical porous carbon material with a favourable charge storage capacity and at the same time finds a high value-added and environment-friendly strategy for disposal and recycling of digested sludge.

## 1. Introduction

With the yearly increase in sewage sludge generation [1], various sewage sludge treatment and disposal technologies have been developed and implemented to protect the environment and human health. Anaerobic digestion is recognized as one of the most widely used treatment methods because it can reduce the amount of sewage sludge for disposal and generate renewable energy by converting biodegradable material into methane [2,3]. Nevertheless, only 20–30% of the organic materials in sewage sludge are mineralized through standard anaerobic digestion technologies [4]. Digested sludge, as the main product of the anaerobic digestion process, still contains considerable amounts of organic compounds [5]. Currently, the most common methods for disposing of digested sludge are land application, combustion and landfilling. Land application poses the risk of damaging the environment due to the existence of heavy metals and hazardous substances in digested sludge [6]. Combustion is difficult because of the high ash and moisture content of digested sludge [7], and landfilling can be very challenging given the dwindling availability of land in developed cities [8]. Therefore, it is necessary and important to explore more cost-effective and environmentally benign reuse of digested sludge.

Supercapacitors are recognized as among the most promising energy storage and supply devices due to their long cycle life, ultrahigh power density and fast charge/discharge process (within seconds) [9]. In general, supercapacitors can be divided into two types according to the energy storage mechanism: pseudo-capacitors and electrochemical double-layer capacitors (EDLCs). Pseudo-capacitors can provide a relatively higher specific capacitance, but their practical applications are limited because of their poor rate capability and cycle stability [10]. EDLCs usually possess excellent charge/discharge cycling stability, which may be ascribed to the electrostatic accumulation of ionic charges at the electrode/electrolyte double-layer interfaces. Carbon-based porous materials have been found to be promising candidates for EDLCs. However, their low energy density may limit practical applications [11]. An effective approach to improving the energy density is to enhance the specific capacitance, which is influenced by the electrochemically accessible surface areas of electrode materials during contact with the electrolyte [12]. For this reason, heteroatom-doped carbon materials with large surface areas and appropriate pore size distribution are desirable for the electrodes in EDLCs. And, introducing defects into carbon materials can also improve the electrochemical performance [13,14]. Lin and co-workers have improved the wettability and electroactive sites of carbon material by creating defects on the side surfaces of few-layered graphene [15].

Recently, researchers have studied the preparation of carbon-based supercapacitor electrodes with sewage sludge as the precursor. For example, Yuan & Dai synthesized carbon electrode material via pyrolysis of sewage sludge and discovered that SiO_2_, an inherent constituent of sewage sludge, acted as a built-in template during the pyrolysis process and facilitated a porous structure [16]. Feng *et al*. reported that the fly-silicon treatment for sewage sludge promoted removal of the ash content and enhanced electrochemical performance [17]. However, from a strategic sustainability perspective, the biodegradable organic matter in the sewage sludge could be first converted into biogas, a renewable energy, before the synthesis process. Thus, compared with the reported sewage sludge-derived supercapacitor electrode material, it would nevertheless be more economic and environmentally sustainable to use digested sludge as a precursor for supercapacitor electrodes.

Given that it contains various O-, N-rich organic compounds and has a porous texture, digested sludge could be considered as a potential precursor for the electrode material of supercapacitors. In this work, for the first time, we prepared a self-doped three-dimensional hierarchical porous carbon material (HPDSC) by facile and sustainable pyrolysis/activation process using digested sludge as the single precursor. The as-prepared HPDSC exhibits a versatile O-, N-doped porous framework with a combination of micropores and meso-/macropores, huge specific surface area, partial graphitization phase and good conductivity. Furthermore, the HPDSC-based electrode shows a desirable specific capacitance, excellent rate performance and outstanding cycling stability, indicating good prospects for HPDSC use in practical applications for high-performance supercapacitor electrodes.

## 2. Material and methods

### 2.1. Materials

As the raw material, the digested sludge was collected from the anaerobic digestion reactor of our laboratory [18] and was stored at −80°C before use. Acetylene black was purchased from Cabot Co., USA. Polytetrafluoroethylene (PTFE) solution (60% suspension in water) was purchased from Aladdin Chemistry Co., Ltd. Potassium hydroxide, hydrochloric acid, hydrofluoric acid and sodium sulfate (Na_2_SO_4_) were of analytical grade and purchased from Sinopharm Chemical Reagent Co., Ltd. All reagents used in this work were used as received without any further purification. Ultrapure water for solution preparation was supplied from a water purification system (Hitech Instrument Co., Shanghai, China).

### 2.2. Synthesis of electrode materials

The obtained digested sludge was vacuum freeze-dried and then pyrolysed at 600 or 800°C under 150 ml min^−1^ of N_2_ flow for 2 h. After natural cooling to ambient temperature in the N_2_ flow, the product was immersed in 20 wt% HF and 1 M HCl aqueous solution to remove impurities. The resultant carbon materials were denoted as DSC-600 and DSC-800, respectively. Then, the DSC-600 was blended with KOH at a mass ratio of 1 : 3 (*W*_DSC-600_/*W*_KOH_) and heated to 800°C for 2 h. After being cleaned and dried, the resultant hierarchical porous digested sludge carbon was denoted as HPDSC.

### 2.3. Material characterization

Scanning electron microscopy (SEM; FEI Nova Nano SEM 450, The Netherlands) and transmission electron microscopy (TEM; JEM 2011, Japan) were used to examine morphologies and microstructures of the as-obtained digested sludge-derived carbon materials. Nitrogen adsorption–desorption isotherm measurements (Quadrasorb evo, Quantachrome Co., USA) were carried out at 77 K to analyse the pore structure. The Brunauer–Emmett–Teller (BET) method and Barrett–Joyner–Halenda model were used to evaluate the specific surface area and pore diameter distribution of carbon materials, respectively. The X-ray diffraction (XRD) patterns (D8 Advance, Bruker Co., Germany) were measured to analyse the crystal structure of carbon materials. Fourier transform infrared spectra (FTIR; Nicolet5700, Thermo Nicolet Co., USA) were used to determine the functional groups of the as-obtained hierarchical porous carbon materials. Raman spectra (Horiba Jobin Yvon Co., France) were measured to analyse the defects in the carbon materials. X-ray photoelectron spectroscopy (XPS) (PHI5000C and PHI5300, Perkin-Elmer Co., USA) was used to investigate the electronic environment of the materials.

### 2.4. Electrochemical measurements

The electrochemical investigations were conducted with a CHI 760E electrochemical workstation (Shanghai Chenhua Instruments Co.) in a three-electrode system, in which a platinum wire served as a counter electrode, Ag/AgCl electrode was used as a reference electrode, and a 0.5 mol l^−1^ Na_2_SO_4_ aqueous solution served as electrolyte. The working electrodes were composed of 10 wt% acetylene black conducting agent, 10 wt% PTFE binder and 80 wt% carbon material (e.g. HPDSC). To prepare the working electrode, a suspension containing the above compounds was loaded on a 1 × 1 cm^2^ nickel foam current collector with a loading capacity of 2–5 mg cm^−2^, and then vacuum dried at 60°C overnight.

Cyclic voltammetry (CV) curves were measured over the potential range of 0–0.8 V versus Ag/AgCl at different scan rates from 5 to 200 mV s^−1^. Galvanostatic charge/discharge (GCD) was conducted galvanostatically at 0.5–11 A g^−1^ with a potential window of 0–0.8 V. The electrochemical impedance spectroscopy (EIS) of the as-obtained digested sludge-derived carbon material was carried out over a frequency range of 0.01 to 100 000 Hz with an alternating current amplitude of 5 mV. The specific capacitance was calculated using the following formula:

2.1 $$C=\frac{I\Delta t}{m\Delta V},$$

where *C* (F g^–1^) is specific capacitance, *I* (A) represents discharge current, Δ*t* (s) is discharge time, *m* (g) is the mass of the carbon materials in the electrode and Δ*V* (V) is the potential window.

## 3. Results and discussion

### 3.1. Structural characteristics

**Figure 1.**
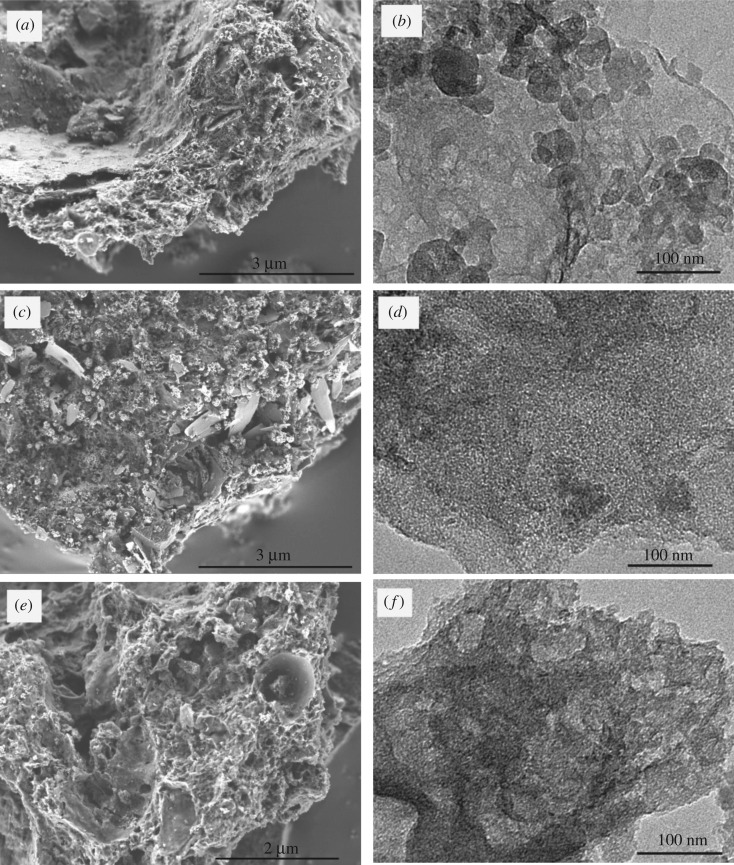
SEM images (*a*,*c*,*e*) and TEM images (*b*,*d*,*f*) of DSC-600 (*a*,*b*), DSC-800 (*c,d*) and HPDSC (*e,f*).

Figure 1 exhibits the SEM and TEM images of as-prepared digested sludge-derived carbon materials. As shown in figure 1*a*, DSC-600 displays irregular porous structures. These porous structures stem from the particular compositions of digested sludge and can be derived from the following three factors: (i) the reserved abundant intrinsic pores in digested sludge after vacuum freezing drying; (ii) SiO_2_ in digested sludge acting as a built-in template, which avoids agglomeration and promotes the generation of the unique pore diameter distribution; and (iii) carbonization and graphitizing of organics in the digested sludge during the pyrolysis calcination process, which results in a rough porous texture. This framework is preserved in as-prepared DSC-800 (digested sludge pyrolysed at 800°C) (figure 1*c*), but it is slightly collapsed, which could be attributed to higher carbonization temperature. Among the three digested sludge-derived carbon materials, HPDSC (figure 1*e*) clearly reveals the most developed three-dimensional network structure and the roughest surface, which indicates KOH activation greatly promotes the formation of pores within the carbon materials. Moreover, TEM images (figure 1*f*) further confirm the interconnected three-dimensional hierarchical porous structure, which can facilitate infinitely fast ion transport and increase the accessible interface area to electrolyte [19].

**Figure 2.**
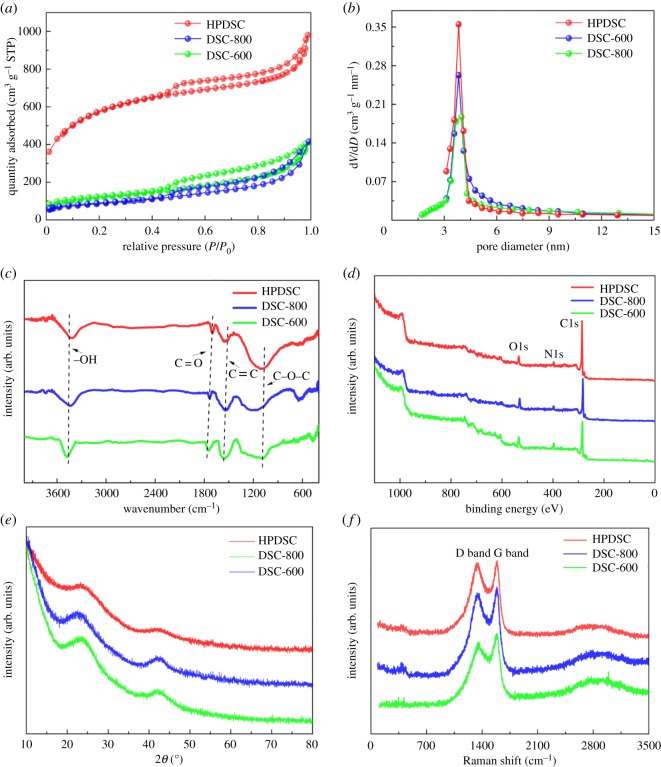
(*a*) Nitrogen adsorption/desorption isotherms and (*b*) corresponding pore size distribution curves of DSCs, (*c*) FTIR of DSCs, (*d*) XPS survey spectra of DSCs, (*e*) XRD patterns of DSCs and (*f*) Raman spectra of DSCs.

To further investigate the detailed textural characteristics of the materials, N_2_ adsorption–desorption isotherms were obtained. All isotherm plots shown in figure 2*a* exhibit typical IV-type curves with an H4 hysteresis loop caused by the capillary condensation in mesopores of the carbon material, suggesting the existence of a considerable number of mesopores in digested sludge-derived carbon materials [20]. The curves exhibit a slight upward tendency when relative pressure is close to 1.0, which could be due to the existence of internal macropores in materials [21]. At a low relative pressure (*P*/*P*_0_ < 0.5), the isotherm plot of HPDSC shows a clear upward trend, suggesting HPDSC is rich in micropores [22]. The result indicates that KOH activation introduces micropores to the hierarchical porous carbon materials.

Figure 2*b* depicts pore diameter distributions determined by N_2_ adsorption for the digested sludge-derived carbon materials and further suggests the intrinsic hierarchical porous structure of these carbon materials. The mesopore structure is predominant in all of the materials because the three curves in figure 2*b* show a peak at 4 nm. Furthermore, the peak of the curve for HPDSC is the sharpest, indicating KOH activation can promote the formation of mesopores in the materials. By combining this observation with the analysis of the nitrogen adsorption isotherm, it can be seen that KOH activation promotes a simultaneous increase in micropore content and the significant development of mesopores in the materials, thereby forming a versatile porous framework with a combination of micropores and meso-/macropores. The three-dimensional hierarchical porous carbon texture helps expose more active functional groups in the carbon skeleton to material surfaces, enhances the specific surface areas of the material and provides more storage sites for electrolyte ions [23].

The relevant mechanisms for KOH activation of carbon mainly occur in three phases: (i) the carbon skeleton is etched via redox reactions between diverse potassium compounds (KOH, K_2_CO_3_ and K_2_O) and carbon, thus generating a large number of micropores; (ii) the gasification of carbon to CO further develops the porosity; and (iii) the generated metallic K can efficiently intercalate into carbon lattices, leading to volumetric expansion of the carbon framework [24]. The chemical reactions can be expressed as follows [25]:

3.1 $$\begin{array}{r} \text{6KOH}+\text{2C}\to \text{2K}+2{\text{K}}_{2}\text{C}{\text{O}}_{3}+3{\text{H}}_{2}, \end{array}$$

3.2 $$\begin{array}{r} {\text{K}}_{2}\text{C}{\text{O}}_{3}+\text{2C}\to \text{2K}+\text{3CO}, \end{array}$$

3.3 $$\begin{array}{r} {\text{K}}_{2}\text{C}{\text{O}}_{3}\to {\text{K}}_{2}\text{O}+\text{C}{\text{O}}_{2}, \end{array}$$

3.4 $$\begin{array}{r} {\text{K}}_{2}\text{O}+\text{C}\to \text{2K}+\text{CO} \end{array}$$

3.5 $$\begin{array}{r} \;\mathrm{and}\;{\text{CO}}_{2}+\text{C}\to \text{2CO}. \end{array}$$

In this activation process, the formed K_2_CO_3_ starts decomposing to K_2_O and CO_2_ at over 700°C and completely disappears at about 800°C [26].

Remarkably, HPDSC has a specific surface area of 2103.6 m^2^ g^−1^, which is the highest among the three materials (the specific surface areas of DSC-800 and DSC-600 are 302.9 m^2^ g^−1^ and 418.8 m^2^ g^−1^, respectively). This high surface area could be due to the thorough KOH activation of carbon materials and makes for a desirable specific capacitance [26].

### 3.2. Chemical composition

The FTIR characterization (figure 2*c*) was performed to analyse the functional groups of the as-prepared carbon materials. All of the spectra show similar vibration absorptions. The characteristic peak located at 3414 cm^−1^ originates from the stretching vibration of the ─OH group [27]. Another peak, at 1698 cm^−1^ in the spectrum, can be ascribed to the stretching vibration of the C═O bond [23]. The broad peak at 1620 cm^−1^ can be attributed to the stretching vibration of the C=C bond, indicating the presence of an sp^2^ hybridized honeycomb lattice [28]. The remaining peak at 1112 cm^−1^ corresponds to the C─O─C bond vibration [29]. The abundance of oxygen-containing groups on the carbon frameworks would provide the essential hydrophilicity property for electrode materials, generate pseudocapacitance and make the materials suitable for use in active electrodes [30].

To further investigate chemical components of the materials, XPS was conducted. As shown in figure 2*d*, all XPS spectra exhibit three distinct peaks at approximately 285 eV, 400 eV and 532 eV, which are caused by the C1s, N1s and O1s orbitals, respectively [24]. The organic matter abundant in O and N in digested sludge is exhibited as O-, N-dopant precursor during this synthesis process. The weak peak intensity of N1s suggests a small quantity of active nitrogen-containing functional groups in the materials, whereas the pronounced peak of O1s shows the three materials contain a large number of surface active, oxygen-containing functional groups. Compared with DSC-600, the contents of nitrogen in DSC-800 decreased due to the vaporization of nitrogenous organic matter at the higher carbonization temperature. The HPDSC has higher carbon content and lower heteroatom (O and N) content than DSC-600, which reflects the removal of a small number of functional groups, including O and N, during the KOH activation process.

**Figure 3.**
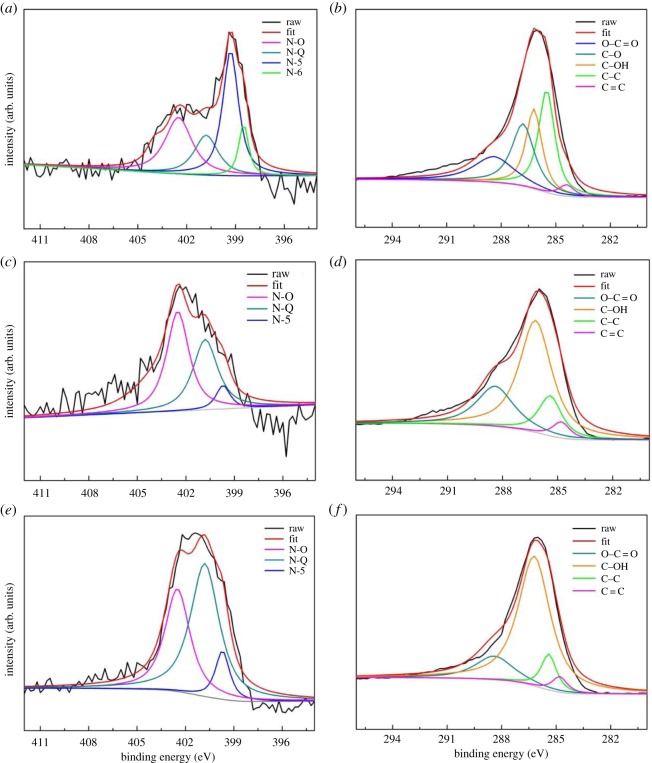
High-resolution N1s XPS spectra of HPDSC (*a*), DSC-800 (*c*) and DSC-600 (*e*). High-resolution C1s XPS spectra of HPDSC (*b*), DSC-800 (*d*) and DSC-600 (*f*).

The N1s core-level spectrum of HPDSC (figure 3*a*) shows four nitrogen peaks, including N-O (oxidized N) at 402.5 eV, N-Q (quaternary N) at 400.8 eV, N-5 (pyrrolic/pyridinic N) at 399.7 eV and N-6 (pyridinic N) at 398.5 eV [31,32]. Among these configurations, N-6 and N-5 with their planar structures were found to be the main sources of pseudocapacitance, whereas N-Q could increase the conductivity of carbon matrixes [33]. The peak fitting analysis shows that the amount of N-5 is significantly high in HPDSC, whereas the most prevalent component in DSC-600 and DSC-800 is N-Q and N-O, respectively. Meanwhile, the N1s spectra (figure 3*c,e*) of DSC-600 and DSC-800 are short of N-6. These results illustrate that the KOH activation is more likely to promote the generation of N-6 and N-5 in the carbon framework of HPDSC, and thus greatly improves its specific capacitance. A detailed study of the C1s peak (figure 3*b*) of HPDSC by deconvolution analysis reveals the coexistence of C=C (284.8 eV), C─C (285.4 eV), C─OH (286.2 eV), C─O (286.8 eV) and O─C═O (288.4 eV) [26]. These results are consistent with the FTIR results.

Figure 2*e* shows the XRD patterns of the digested sludge-derived carbon materials. It can be observed clearly that all of these materials show two peaks at 2*θ* = 24° and 43°, which can be attributed to the (002) and (101) facets of amorphous carbon, respectively, and indicates the materials are partially crystalline [34]. The intensities of the two peaks for HPDSC are slightly reduced, suggesting KOH activation at 800°C partially destroys the crystal structure. This is further confirmed by the Raman spectra. Remarkably, the intensity increases sharply at the low-angle scattering peak, indicating the presence of a good deal of micropores [35]. These indications are in accordance with the results from the BET analyses.

The Raman spectra for the digested sludge-derived carbon materials are illustrated in figure 2*f*. All the curves exhibit two prominent peaks at 1340 and 1590 cm^−1^, which are caused by the characteristic D and G bands of the materials, respectively [36]. The D band reflects the degree of defects in carbon, whereas the G band corresponds to the degree to which the materials are graphitized [37,38]. The strong intensity of G bands for the materials indicates their good graphite-like structure. The *I*_D_/*I*_G_ values (the intensity ratio of D band to G band) of DSC-600, DSC-800 and HPDSC are 0.86, 0.91 and 0.98, respectively. These results suggest that higher carbonization temperature and the KOH activation process promote the production of defects from the ideal graphitic lattice, which is in accordance with the XRD results.

### 3.3. Electrochemical properties

**Figure 4.**
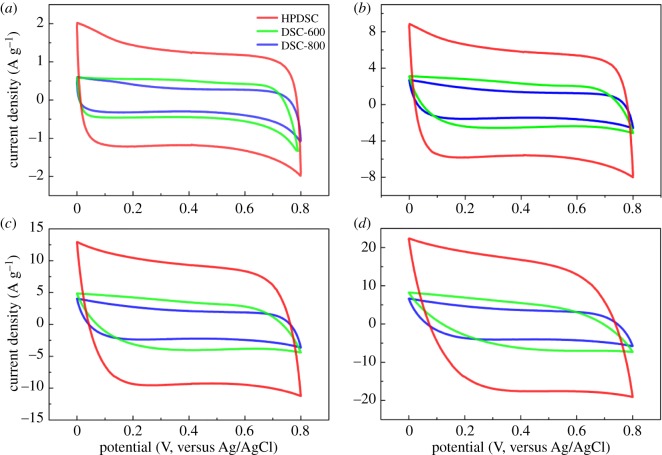
CV measurements of HPDSC, DSC-800 and DSC-600 in 0.5 M Na_2_SO_4_ aqueous solution over a potential range from 0 to 0.8 V at a scan rate of (*a*) 5 mV s^−1^, (*b*) 30 mV s^−1^, (*c*) 50 mV s^−1^ and (*d*) 100 mV s^−1^.

Given the existence of interconnected, hierarchically porous structure with suitable pore size, O-, N-doped framework, reinforced hydrophilicity and high surface area, the digested sludge-derived carbon materials are expected to be promising candidates for use in supercapacitor electrodes. To explore the electrochemical properties of these materials, CV measurement was performed in a three-electrode system in 0.5 M Na_2_SO_4_ aqueous solutions at room temperature (figure 4). Apparently, at the same potential scan rates, all CV plots exhibit quasi-rectangular-like mirror image characteristics from 0 to 0.8 V, which can be attributed to the similar chemical composition of these materials and indicates that the capacitance originated mainly from the electronic double-layer capacitor.

The loop area of DSC-800 is slightly smaller than that of DSC-600, suggesting the higher carbonization temperature is not required for high capacitance. Compared with DSC-600, HPDSC exhibits much larger loop areas with bigger plateau currents, which indicates KOH activation can promote dramatically increased capacitance. By KOH activation, the specific surface area of the carbon electrode material increases from 418.8 m^2^ g^−1^ for DSC-600 to 2103.6 m^2^ g^−1^ for HPDSC, which means there is a larger accessible surface area for electrolyte ion accumulation. Moreover, KOH activation promotes a simultaneous increase in micropore and mesopore content, forming an advanced three-dimensional hierarchical porous texture with abundant mesopores, moderate micropores and macropores. Micropores can vastly increase the specific surface area of carbon materials and thus provide more storage sites for electrolyte ions [39]. Mesopores are extremely important for providing an expedited and efficient ion transfer pathway, thus improving the infinitely fast ion transport and further heightening electrolyte accessibility to the micropores area [40]. Macropores act as an ion reservoir and reduce the ion diffusion distance [41]. To sum up, KOH activation could significantly enlarge the specific capacitance of porous carbon materials by greatly expanding the specific surface area and forming a desirable porous structure, and HPDSC has the best potential as an electrode material among the materials studied.

**Figure 5.**
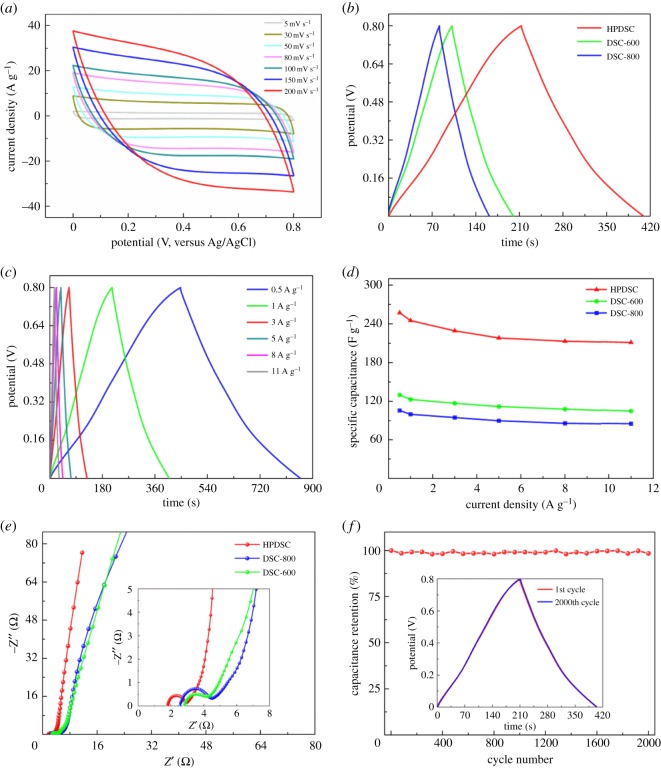
Electrochemical performance characteristics measured in a three-electrode system in 0.5 M Na_2_SO_4_ electrolyte. (*a*) CV curves of HPDSC at different scan rates varying from 5 to 200 mV s^−1^, (*b*) charge–discharge curves of DSCs at a current density of 1 A g^−1^, (*c*) charge–discharge curves of HPDSC at different current densities, (*d*) specific capacitances of DSCs at different current densities, (*e*) EIS spectra (inset: magnified 0–8 Ω region) under the influence of an AC voltage of 5 mV and (*f*) cyclic stability of HPDSC at a charge–discharge current density of 1 A g^−1^ for 2000 cycles (inset: GCD plots of HPDSC after 1st and 2000th charge–discharge cycles).

Figure 5*a* demonstrates the CV plots of the HPDSC at different potential scan rates. It is clear that loop area increases as the scanning rate increases. The CV plots still retain the roughly rectangular shape with little deformation at scan rates up to100 mV s^−1^, suggesting low internal resistance, rapid electrolyte ion diffusion kinetics and good rate capability. The gradual tilt of CV plots at higher scan rates of 150 and 200 mV s^−1^ is likely due to the unavoidable resistance of electrode and electrolyte.

To further evaluate the capacitance performance and calculate the specific capacitance of the materials, GCD experiments were conducted at different current densities. Figure 5*b* shows the GCD curves of the three carbon nanomaterials at a current density of 1.0 A g^−1^. All the GCD curves in figure 5*b* exhibit linear and symmetrical triangular shapes, indicating the electrodes made with carbon nanomaterials have excellent double-layer capacitor behaviours and electrochemical reversibility during the charge and discharge process. Additionally, there is a small voltage drop where the discharge curves start, which originates from the unavoidable internal resistance of the carbon electrode materials. At a current density of 1.0 A g^−1^, the calculated specific capacitances of DSC-600, DSC-800 and DSHPC are 123 F g^−1^, 100 F g^−1^ and 245 F g^−1^, respectively. These results suggest that the capacitance properties could be further reinforced by KOH activation and that electrodes made with HPDSC possess superior capacitance behaviour. This accords well with the conclusion of CV measurement.

GCD curves for HPDSC at different current densities from 0.5 to 11 A g^−1^ are shown in figure 5*c*. It is notable that a symmetric triangular shape with small IR drops is still retained at the high current density of 11 A g^−1^. This result shows that the HPDSC electrode has good rate capability, which promotes the fast charge/discharge of supercapacitors. As shown in figure 5*d*, the calculated specific capacitance for the HPDSC is 257 F g^−1^, 245 F g^−1^, 229 F g^−1^, 218 F g^−1^, 213 F g^−1^ and 211 F g^−1^ at the current densities of 0.5 A g^−1^, 1 A g^−1^, 3 A g^−1^, 5 A g^−1^, 8 A g^−1^ and 11 A g^−1^, respectively, with the specific capacitance retention as high as 82.1%. The decrease of specific capacitance is due mainly to the insufficient electrolyte ion diffusion kinetics of HPDSC in Na_2_SO_4_ electrolyte; that is, electrolyte ions fail to efficiently access the porous electrode interface with the increase of current density.

To further understand the capacitive behaviours of these materials, the EIS test was performed with an AC modulation of 5 mV in a frequency range of 10^−2^–10^5^ Hz. Figure 5*e* shows the Nyquist plots for the HPDSC, DSC-800 and DSC-600 electrodes in the 0.5 M Na_2_SO_4_ electrolyte. All three EIS spectra have a similar shape, with a semicircle at high-frequency region and a straight line in the intermediate region. It is known that the semicircle can be attributed to the charge-transfer resistance (CTR) from the interface of the electrode to electrolyte, which is caused by Faradic reactions and EDLC [42]. Generally, the bigger the semicircle diameter, the larger the impedance will be. As shown in figure 5*e*, the HPDSC electrode's Nyquist plot has the smallest semicircle diameter, meaning it has the lowest CTR at the active material/electrolyte interface. This indicates that fast charge transportation could be achieved with the electrode made with HPDSC. The 45° straight line in the intermediate frequency region corresponds to the Warburg region, which represents ion diffusion into hierarchical porous carbon electrode materials [43]. The EIS spectrum of the HPDSC electrode exhibits the shortest Warburg region, indicating that the fastest ion diffusion occurs in the HPDSC electrode. All the EIS spectra possess a near-vertical line over the low frequency range, suggesting good capacitor behaviours of digested sludge-derived carbon materials, especially for HPDSC [44]. Furthermore, the *x*-intercept in the high frequencies indicates the equivalent series resistance (ESR), which derives mainly from the inner resistance of active materials, electrolyte ionic resistance and contact resistance between the current collector and the electrode [45]. The ESR values of HPDSC, DSC-800 and DSC-600 are 1.80 Ω, 2.55 Ω and 2.71 Ω, respectively, suggesting HPDSC possesses the optimal conductivity as a result of the inherent N-Q, partial graphitization phase and the interconnected three-dimensional porous structure.

Cyclic stability is another significant factor determining the practical use of carbon electrode materials. Figure 5*f* shows the small fluctuation in specific capacitance with cycle number for the HPDSC electrode, which was researched by GCD at a current density of 1 A g^−1^ in Na_2_SO_4_ electrolyte. After 2000 cycles, the specific capacitance of the HPDSC electrode amounts to 241 F g^−1^ with 98.4% retention of initial capacitance (245 F g^−1^), which is superior to the electrochemical performance of three-dimensional graphene material with a specific capacitance of 200 F g^−1^ in aqueous electrolyte and 86.2% retention after 5000 cycles [46]. These results indicate high cycle stability of the HPDSC electrode and a promising future for its use in supercapacitors. HPDSC's high cycling stability can also be confirmed by the GCD curves (inset of figure 5*f*) before and after 2000 charging/discharging processes, which overlap almost perfectly. This further highlights the application potential of HPDSC electrodes in excellent-performance supercapacitors.

Thus, the excellent capacitance performance of the HPDSC can be attributed to the following properties: (i) the obtained interconnected three-dimensional hierarchical porous structure with moderate pore size distribution, which facilitates rapid electrolyte ion diffusion during the process of charges/discharges; (ii) the large accessible surface area, which provides more storage sites for electrolyte ions; (iii) the high hydrophilicity caused by abundant O-doped surface functional groups; and (iv) the excellent electrical conductivity improved by the presence of doped N atoms in the carbon framework and the partial graphitization phase of HPDSC.

## 4. Conclusion

Self-doped three-dimensional hierarchical porous carbon material for high-performance supercapacitor electrodes was synthesized successfully from digested sludge via facile and sustainable pyrolysis/activation process for the first time. An interconnected O-, N-doped three-dimensional porous framework with appropriate pore diameter distribution and huge specific surface area enable the material to exhibit excellent electrochemical performance, such as desirable specific capacitance, remarkable rate performance and outstanding cycle stability. The unique qualities of the HPDSC and its excellent electrochemical performance mainly originated from the particular compositions of digested sludge. This protocol offers a facile method for high value-added and environment-friendly reuse of digested sludge, while discovering a new pathway for economic and sustainable preparation of high-performance supercapacitor electrode materials from pollutants. Furthermore, the advanced carbon materials prepared in this work could be used in other practical applications, such as adsorbents and catalyst carriers.

## Data accessibility

This article does not contain any additional data.
